# Mental representation and motor imagery training

**DOI:** 10.3389/fnhum.2014.00328

**Published:** 2014-05-22

**Authors:** Thomas Schack, Kai Essig, Cornelia Frank, Dirk Koester

**Affiliations:** Neurocognition and Action–Biomechanics Research Group, Center of Excellence “Cognitive Interaction Technology”, Research Institute for Cognition and Robotics, Bielefeld UniversityBielefeld, Germany

**Keywords:** mental representation, mental imagery, motor imagery, mental simulation, basic action concepts

## Abstract

Research in sports, dance and rehabilitation has shown that basic action concepts (BACs) are fundamental building blocks of mental action representations. BACs are based on chunked body postures related to common functions for realizing action goals. In this paper, we outline issues in research methodology and an experimental method, the *structural dimensional analysis of mental representation* (SDA-M), to assess action-relevant representational structures that reflect the organization of BACs. The SDA-M reveals a strong relationship between cognitive representation and performance if complex actions are performed. We show how the SDA-M can improve motor imagery training and how it contributes to our understanding of coaching processes. The SDA-M capitalizes on the objective measurement of individual mental movement representations before training and the integration of these results into the motor imagery training. Such motor imagery training based on mental representations (MTMR) has been applied successfully in professional sports such as golf, volleyball, gymnastics, windsurfing, and recently in the rehabilitation of patients who have suffered a stroke.

The representation and simulation of motor acts has a long and varied history in psychology and movement science. Johann Friedrich Herbart related movements to perceptual effects as early as 1825 and proposed that the imagery of perceptual effects can elicit the related movements (p. 464). William James (1890, p. 526) wrote some decades later “that every representation of a movement awakens in some degree the actual movement…”. These and other approaches of an ideomotor understanding of human action felt out of fashion in the era of behaviorism (see Shin et al., 2010 for a review). However, around 100 years later they became an important reference point for many experimental approaches, for example, *ideomotor action* (e.g., Knuf et al., 2001; Kunde, 2001; Koch et al., 2004; Kunde et al., 2004), *common coding* (Prinz, 1987, 1997), *anticipative behavioral control* (Hoffmann, 1993; Hoffmann et al., 2004), *theory of event coding* (Hommel et al., 2001) and *cognitive architecture of action approach* (Schack and Mechsner, 2006; Schack and Ritter, 2009). These approaches underline the goal-directedness of human behavior (termed “motor cognition” by Jeannerod, 2006, p. v) and are considered an alternative to non-cognitive approaches to human movement.

These new perceptual-cognitive perspectives emphasize the goal-directedness property of actions, the importance of anticipated perceptual effects, the crucial role of mental representations in action control and the functional role of mental simulation for planning and performing voluntary movements with the help of structured cognitive representations of action effects (Hommel et al., 2001; Mechsner et al., 2001; Schack and Mechsner, 2006; Hoffmann et al., 2007). Furthermore, skillful coordination occurs if appropriate mental representations of the motor task and action goals are constructed, because cognitive representations govern the tuning of motor commands and muscular activity patterns. In fact, this perceptual-cognitive approach to movement control is reminiscent of some classical ideas in psychology, such as the “ideomotor” approach adopted by Lotze (1852) and James (1890) in the 19th century and the theoretical studies of movement construction by Bernstein (Bernštejn) (1947, English translation 1967) in the 20th century. Although this perspective never disappeared, it was obscured by the dominant and competitive perspectives of cognitive and dynamical systems approaches to motor control.

Whereas dynamical systems in principle try to explain biological movements without alluding to cognitive levels (or internal models), Bernstein (Bernštejn) (1947) envisaged a complex architecture of human movement control ranging from “low” levels corresponding to involuntary movements, up to “high” cognitive levels that can be thought of as concepts. The second “lowest” level corresponds to synergistic processing, and this level has often been referred to in dynamical systems approaches (e.g., Wolpert et al., 1995; Ijspeert et al., 2002). We note that spinal (e.g., D’Avella and Bizzi, 1998; Poppele and Bosco, 2003) and some muscle synergies do not always require input from higher cognitive levels (e.g., Debicki and Gribble, 2005). Such aspects of involuntary movements are often addressed in sensorimotor models of motor control (e.g., Kawato, 1999; Todorov, 2004). These processes run mostly automatically but can reach conscious levels if attention is directed towards them. In stark contrast to “low” levels, our understanding of *cognitive* movement control is far less known. Therefore, in this paper, we have focused on “higher” (i.e., cognitive) levels of human motor control (perceptual-cognitive approach), and suggest that cognitive representations should be differentiated from cognitive *control* of movements (cf. Schack, 2004a, b, 2010). That is, our approach is more closely related to cognitive approaches for motor control such as Schmidt’s classical schema theory^1^ (Schmidt, 1975) and is in competition with dynamical system approaches. However, we do not suggest that motor control, or even voluntary movements, are solely controlled from a cognitive level. Involuntary motor control (e.g., reflexes and postural control) are also critically important as sensorimotor loops. We will discuss how cognitive levels of movement representation and control can be measured and used for (training) interventions. Among the key issues are how structured mental representations can arise during motor skill acquisition and how these representations attain a functional role in motor learning. Related questions concern the role of cognitive representations in motor imagery training and, prospectively, whether motor imagery can be applied to technical platforms and robotics.

## Mental representation

It is a well established idea in cognitive psychology and indeed it has received growing acceptance in the fields of motor control and sport psychology that actions are mentally represented in functional terms as a combination of the executed action and the intended or observed effects (Prinz, 1997; Hommel et al., 2001; Knuf et al., 2001; Koch et al., 2004; Jeannerod, 2006, p. 165). The link between movements and perceptual effects is bi-directional and is thought to be stored hierarchically in long-term memory (LTM). Such movement representations are necessary because complex movements are highly unlikely to rely solely on online calculation due to human resource limitations. Rosenbaum and co-workers (Rosenbaum et al., 2007) demonstrated that movements can be understood as a serial and functional order of goal-related body postures, or goal postures, and their transitional states. That is, movements can be understood as the changes between body postures. Whereas body postures (keyframes) are represented in detail, the interframes (i.e., the movements between body postures) contain only differences between two successive keyframes. The better the order formation within cognitive movement representations, the more easily information can be accessed and retrieved (Schack and Ritter, 2009). This leads to increased motor execution performance, which reduces the amount of attention required for successful performance (Beilock et al., 2002; Raab and Johnson, 2007; Land et al., 2014). The nodes within such networks of movement representation contain functional subunits or building blocks that relate motor actions and associated perceptual effects.

Researchers from different fields, such as cognitive psychology, cognitive robotics and sport psychology (Schack, 2004a, b; Schack and Mechsner, 2006; Schack and Ritter, 2009, 2013; Tenenbaum et al., 2009; Maycock et al., 2010), have provided evidence for so-called basic action concepts (BACs) in the control of human movements. Analogous to the well-established notion of basic object concepts (Rosch, 1978), BACs are the mental counterparts of functional elementary components of complex movements. They can be thought of as the cognitive chunking of body postures and movement events concerning common functions in realizing action goals. BACs do not refer to behavior-related invariant properties of objects, as in the case in basic object concepts, but to perception-linked invariant properties of movements. According to the cognitive action architecture approach (Schack, 2010), mental representations are thought to comprise of such representational units (i.e., BACs) and their structural composition in relation to one another.

To investigate representational networks of BACs, the *structural dimensional analysis of mental representation* (SDA-M) method was developed by Schack (2004a). Various methods facilitate the study of knowledge-based mental representations of movements in LTM (for an overview, see Hodges et al., 2007). However, most of them focus on explicit knowledge and are non-experimental (e.g., interviews, questionnaires, paper-and-pencil tests). As an experimental method that avoids introspective statements, Schack (2004a, 2010) introduced the SDA-M method. This method provides psychometric data on mental representations of complex movements and as such permits investigating the status and change of structures of mental movement representations.

In detail, the SDA-M (Schack, 2010) maps mental representations as integrated networks of BACs across both individuals and groups, by providing information on relational structures in a given set of concepts with respect to goal-oriented actions. The internal grouping of conceptual units (i.e., the clustering of BACs) delineates the structure of the knowledge representation of a certain movement. While mental representation structure refers to the relation and the grouping of BACs in LTM, learning can be considered as the modification of the mental representation structure over time. That is, mental representation of complex movements can be measured by the SDA-M method.

The SDA-M consists of four steps (for further details, see Schack, 2012). First, a split procedure involving a multiple sorting task (pair-wise comparisons) delivers a distance scaling between BACs of a suitably predetermined set. Specifically, during this procedure, one concept of a given set of BACs is permanently displayed on a computer screen (anchor concept) and all other concepts are compared to that anchor concept successively. Participants have to decide whether the two given concepts are related to each other during movement execution. The procedure continues, until all concepts have been compared to all other concepts. Second, a hierarchical cluster analysis is used to transform the set of BACs into a hierarchical structure. Third, a factor analysis reveals the dimensions in this structured set of BACs, and fourth, the cluster solutions are tested for invariance within or between groups.

**Figure 1:**
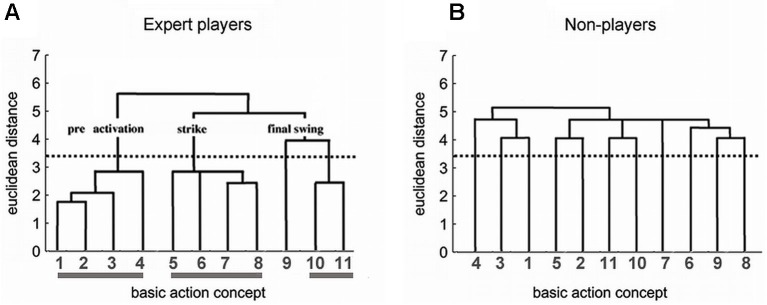
**Dendrograms for the experts (A) and non-players (B) based on the hierarchical cluster analysis of BACs in the tennis serve**. The horizontally aligned numbers denote the BACs (for the code, see text), the vertical numbers, the euclidean distances. For every group, it holds *n* = 11; *p* = 0.05; *d*_crit_ = 3.46. A tennis serve consists of three distinct phases, each of which fulfills distinct functional and biomechanical demands. First, in the pre-activation phase, body and ball are brought into position, and tension energy is provided to prepare the strike. The following BACs were identified: (1) ball throw, (2) forward movement of the pelvis, (3) bending the knees, and (4) bending the elbow. Second, in the strike phase, energy is conveyed to the ball. The following BACs were identified: (5) frontal upper body rotation, (6) racket acceleration, (7) whole body stretch motion, and (8) hitting point. Third, in the final swing phase, the body is prevented from falling, and the racket movement is decelerated after the strike. The following BACs were identified: (9) wrist flap, (10) forward bending of the body, and (11) racket follow through.

As a result, one obtains the individual partitioning of the BACs in hierarchical tree-like structures, the so-called dendrograms (see Figure 1). Cluster solutions are calculated for all individual participants and for the whole group. Each cluster solution is established by determining a critical Euclidean distance *d*_crit_ (marked by the dotted horizontal line in Figure 1). The critical value *d*_crit_ depends on the number of concepts. All junctures below the value *d*_crit_ are considered related, while the junctures above this value are considered unrelated. This results in a cluster solution. In an optimal structure, the resulting cluster solution represents the functional phases of the movement.

A good example to investigate the mental representation structures of a complex movement on different levels of expertise is the tennis serve (Schack and Mechsner, 2006). For a tennis serve, not only many degrees of freedom have to be controlled in the musculoskeletal system, but also the correct movement execution depends considerably on training and expertise. On the other hand, it is a finite and recognizable action pattern of which the overall structure is well defined by biomechanical demands.

The expert group in that study consisted of 11 male tennis players (mean age, 24 ± 3.7 years) from the upper German leagues who were ranked between places 15 and 500 in the German men’s rankings. The non-player group were 11 males (mean age, 24 ± 6.7 years) with virtually no experience of the game (maximum 5 h) and had never had any tennis lessons.

The single BACs and the adequate functional organization of the tennis serve were characterized in advance in collaboration with non-players, athletes with different levels of expertise, and coaches. Photographs of the tennis sub-movements were presented to experts and non-players together with linguistic markers of varying generality. The picture-word combination which took the shortest time to judge its appropriateness was chosen, in analogy to classical methods (Rosch, 1978).

Each BAC was characterized by a set of closely interconnected sensory and functional features. For example, BAC 7 (whole body stretch motion) is functionally related to providing energy to the ball, transforming tension into swing, stretching but remaining stable. Afferent sensory features of the corresponding sub-movement that allow monitoring of the initial conditions are bent knees, tilted shoulder axis, and body weight on the left foot. Re-afferent sensory features that allow monitoring of whether the functional demands of the sub-movements have been addressed successfully are muscles stretched and under tension, proprioceptive and, finally, perhaps visual perception of the swinging arm and ball in view.

Figure 1A depicts the dendrogram for the experts. Their cognitive structure was very similar to an optimal cluster solution and matches the functional and biomechanical demands of the tennis serve. The three functional phases (i.e., pre-activation, strike, and final swing) form clearly separated clusters in the dendrogram. An invariance analysis (step four of the SDA-M) confirmed this interpretation. There was no significant difference between the cognitive BAC framework in experts and the biomechanical demand structure of the movement. In contrast, the clustering of the BACs in the dendrogram of the non-players (Figure 1B) did not mirror the functionally and biomechanically demanded phases so well. The BACs were less clearly grouped, with no close neighborhoods, and the partial clusters largely failed to attain significance. The average novice structure, however, deferred significantly from the optimal cluster solution (cf. Schack and Mechsner, 2006). That is, in experts, these representational frameworks were organized in a distinctive hierarchical tree-like structure, were remarkably similar between individuals, and were well-matched with the functional and biomechanical demands of the task. In contrast, action representations in low-level players and novices were organized less hierarchically, were more variable among persons, and were less well-matched with functional and biomechanical demands.

More generally, if two BACs are frequently classified by participants as being “functionally related” during the split procedure, these BACs are characterized by a small Euclidean distance which is reflected in a low projection of the BACs on the vertical axis in the dendrogram (e.g., BACs 1 and 2 in Figure 1A). If two BACs are not judged to be “functionally related”, the Euclidean distance is larger and the projection of the two BACs is high in the tree diagram (e.g., BACs 9 and 10 in Figure 1A).

In order to measure the inter-individual or inter-group differences between representation structures, a structural invariance measure λ is determined based on (1) the number of constructed clusters of the pair-wise cluster solutions; (2) the number of concepts within the constructed clusters; and (3) the average quantities of the constructed clusters. The invariance measure λ ranges from 0 (no similarity at all) to 1 (tree diagrams are identical). Two cluster solutions (or representation structures) are considered to be invariant (i.e., the same) if λ > λ_crit_ = 0.68 (which corresponds to a significance level of *α* = 0.05; for more detailed information, see Schack, 2010, 2012).

Furthermore, as shown in a volleyball study (Schack, 2004b), these mental representation structures are position- and task-dependent. Such representation structures are the outcome of an increasing, effort-reducing formation of order in LTM. With increasing expertise, the representation of the movement corresponds more and more to its topological (spatiotemporal) structure. Accordingly, movement control becomes possible by representing the anticipated perceptual effects of the movement and comparing them with incoming perceptual effects.

Accordingly, the structure of cognitive representations in LTM is also relevant for perception and visuomotor control in motor action. But little is known about the relationship of cognitive representations and visuomotor control for complex movements. Therefore, in a recent study we investigated whether cognitive representations of complex movements influence (unconscious) visual perception (Güldenpenning et al., 2011). Novices and skilled high-jump athletes were shown to differ in that only skilled athletes have a functionally structured, cognitive representation of the high jump movement (Fosbury flop). Both groups were asked to classify pictures of body postures of the high jump movement. In a so-called priming paradigm, each of these picture presentations were preceded by another picture of a high jump body posture that could not be perceived consciously. Participants had to classify whether the second pictures in each trial showed a body posture from the approach or from the flight phase. Importantly, the two pictures in each trial could differ with regard to the shown movement phase but also in temporal order. That is, both pictures could reflect the natural order within or between movement phases or, alternatively, they could be presented in a reversed order (e.g., flight before approach). We found a main effect of temporal order for skilled athletes, that is, faster reaction times for picture pairs that reflected the natural movement order as opposed to the reversed movement order. Novices showed a qualitatively different data pattern which was in line with superficial processing of visual features unrelated to the high jump movement. These results suggest that the structure of cognitive movement representations modulates the visual processing of body postures. Temporal information seems to be an important dimension of such representations (cf. also Güldenpenning et al., 2013) and can be processed automatically as the extraction of temporal order information required unconscious processing of (one of) the pictures.

Based on these and many other studies (e.g., Haggard and Wolpert, 2005; Giummarra et al., 2007; Bläsing et al., 2010b), we argue that major interfaces in the architecture of movement are cognitive in nature (without fully denying the relevance of automated processes such as reflexes or postural control of the whole body). Such a perspective does not view the motor system as being distinct from cognition. Instead, it considers both conscious and automatized processes of movement organization to be based functionally on cognitive representation structures. This does not ignore the significance of emotional or motivational processes; it simply puts them aside in order to focus on the cognitive architecture of movement (Schack and Ritter, 2009). In the next sections we will consider how mental representations change during motor imagery and motor learning.

## Mental representation and learning

Differences in the mental representation structure between novices, intermediates, and experts (Schack and Mechsner, 2006; Bläsing et al., 2009) suggest that the structure of mental representations of complex movements changes with improvements in the skill-level. More specifically, the structure of the mental representation of a given complex movement might develop towards the functional structure of an expert over the course of practice. Therefore, a novice’s unstructured representation of a movement is thought to develop into a more structured representation during motor learning. Accordingly, we assume learning to be a product of modifying the mediating structure among the BACs (see Schack, 2004a).

To the best of our knowledge, there are only a few studies examining how mental representation structures develop during practice. As it seems crucial to learn more about whether and when changes in mental representations occur and how they develop during learning, we examined structural changes in mental representations of a complex movement during early skill acquisition (Frank et al., 2013). The acquisition of the golf putting movement was investigated in a group of novice golfers. After a 3 day period of practice with the task, the mental representation of the practice group was compared to that of a control group. As expected, the mental representation structure showed functional changes (i.e., functional clusters in the group’s dendrogram) in the practice group along with performance improvement while no such changes were observed in the control group.

**Figure 2:**
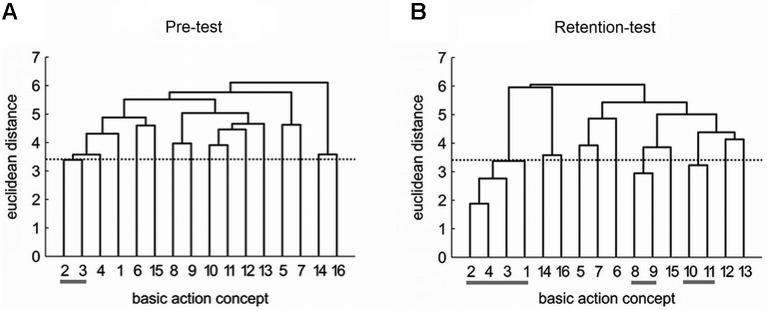
**Mean group dendrograms of the practice group (*n* = 12) for the golf putt at (A) pre-test and (B) retention-test.** The numbers on the *x*-axis relate to the BAC number, the numbers on the *y*-axis display euclidean distances. The lower the link between related BACs, the lower is the euclidean distance. The horizontal dotted line marks *d*_crit_ for a given *α*-level (*d*_crit_ = 3.41; *α* = 0.05); links between BACs above this line are considered unrelated; horizontal gray lines below BAC numbers mark clusters. BACs: (1) shoulders parallel to target line, (2) align club face square to target line, (3) grip check, (4) look to the hole, (5) rotate shoulders away from the ball, (6) keep arms-shoulder triangle, (7) smooth transition, (8) rotate shoulders towards the ball, (9) accelerate club, (10) impact with the ball, (11) club face square to target line at impact, (12) follow-through, (13) rotate shoulders through the ball, (14) decelerate club, (15) direct clubhead to planned position, and (16) look to the outcome [Reprinted from Frank et al., 2013, with permission].

Specifically, the mental representation structure of the practice group changed over the course of practice from pre-test (Figure 2A) to retention-test (Figure 2B) and became more similar to an expert structure. As shown in Figure 2, the practice group’s mean dendrogram revealed an increased number of functional clusters during retention-test, with BACs being clustered into three functional units relating to distinct movement phases (i.e., movement preparation, the forward swing, and the impact phase). In contrast, no changes were evident in the mental representation structure of the control group which did not execute the putt at all. These findings suggest that order formation of action-related knowledge plays a significant role during motor learning, presumably, for the development of movement expertise. Further investigations from a number of different activities (e.g., golf, soccer, wind surfing, volleyball, gymnastics, and dancing) also support the functional relation between mental representation structures and performance and expertise (Schack, 2004a; Schack and Bar-Eli, 2007; Schack and Hackfort, 2007; Bläsing et al., 2009, 2010a; Velentzas et al., 2011).

## New directions: application of SDA-M in motor imagery training

Studies in the first half of the 20th century indicate that performing mental tasks leads to subsequent performance improvements (Sackett, 1935; see also Driskell et al., 1994). Generally, imagery refers to a collection of abilities, including, for example, visual imagery, kinesthetic imagery, imagery of movements or combinations of imagery modalities (e.g., Callow and Hardy, 2005; Holmes, 2007; Roberts et al., 2008) and there continues to be no consensus on the definitions of imagery. In sports, the subject of imagery is traditionally related to movement (i.e., motor imagery, cf. Jeannerod, 1994) and the main aim of motor imagery is to enhance specific motor actions (cf. Boschker, 2001). Studies have shown that specific training can increase the amount and the efficiency of kinaesthetic imagery and enhance the imagery of kinaesthetic sensations, making images more complex and vivid (Nordin and Cumming, 2007; Golomer et al., 2008). Motor imagery is a cognitive tool strategically used by athletes for learning and optimizing their specific movement tasks. Dancers, for example, use motor imagery to exercise the memorization of long sequences and to improve movement quality in terms of spatiotemporal adaptation and artistic expression. Whereas mental practice or mental training encompass further techniques such as self-talk, goal setting or attention focusing, we refer by “motor imagery training” to the act of repeatedly imagining a movement without executing the movement and with the primary intent of acquiring and optimizing motor skills (for an overview see Morris et al., 2005).

Various theories have been used to explain the effects of motor imagery training (or mental practice, e.g., Heuer, 1985; Driskell et al., 1994). The major scientific models largely differentiate physiologically peripheral (neuromuscular) effects and central effects (e.g., symbolic codes or programs). It has been suggested that motor imagery is based on simulation processes that recruit motor representations, and that imagery, observation and execution of movements share a major part of their neural correlates (so-called functional equivalence, Jeannerod, 1995, 2001; Kosslyn et al., 2001). Furthermore, it has been shown that motor imagery involves internal motor attention processes and states of high concentration (Munzert et al., 2008).

Importantly, the *perceptual-cognitive hypothesis* opens up a new explanation for the effects of motor imagery training. This hypothesis is derived from the theory of ideomotor action (Knuf et al., 2001; Koch et al., 2004) and is in line with current neurophysiological findings (Jeannerod, 1995, 2004). The perceptual-cognitive hypothesis posits a representational system in which strong cognitive representation units (nodes) are linked to perceptual representations (e.g., kinesthetic, optical, or acoustic effect codes). Because they possess a spatiotemporal structure, these representations can be related directly to movements. This makes additional motor, spatial-pictorial, or symbolic representations unnecessary for movement control (see Heuer, 1985). Another basic assumption of the perceptual-cognitive model is that imagining a movement and performing it are based on the same representations (Jeannerod, 1995, 2004), which can explain the effectiveness of motor imagery training. Mental simulations of movement may strengthen links between cognitive representation of intermediate states of that movement and the accompanying perceptual effect codes. At the same time, interfering perceptual inputs will be inhibited.

This makes the SDA-M method proposed here directly relevant for developing new forms of motor imagery training (cf. also Cooley et al., 2013). One central question in sport psychology has been the question of how to best tailor and deliver motor imagery training such that it is most effective in enhancing an athlete’s performance and in promoting learning. The main disadvantage of traditional procedures is that they try to optimize performance without taking the athlete’s mental technique representation into account (i.e., they are representation-blind). If the movement’s cognitive representation has structural gaps or errors, these will tend to be stabilized rather than overcome by repeated practice. An alternative method here is to measure the mental representation of the movement before motor imagery training and then integrate these results into the training. Thus, similarly to the finding that imagery tailored to the individual is more promising compared to standardized procedures (for an overview, see Schuster et al., 2011), we suggest that the individual’s prerequisites should be considered when applying motor imagery training. As opposed to more subjective measures such as interview techniques, the SDA-M method is an objective measure of BACs and their relations (i.e., mental representation structure). As such, the SDA-M serves to tailor imagery content of subsequent mental practice according the individual’s cognitive status. This Motor imagery Training based on Mental Representations (MTMR^2^) has now been applied successfully for several years in professional sports such as golf, volleyball (Schack, 2004b), gymnastics (Schack and Heinen, 2000; Heinen et al., 2002), and windsurfing (Schack and Hackfort, 2007).

**Figure 3:**
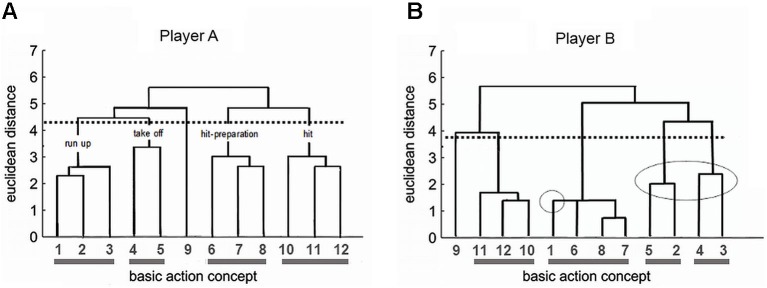
**Memory profile of the spike in player A’s (A) and player B’s (B) movement memory.** BACs: (1) taking arms back, (2) stamp step, (3) bending knees and trunk, (4) swinging both arms forward, (5) extending legs, (6) body arching, (7) spiking arm back, (8) high elbow, (9) glance toward opponent’s block, (10) spike emphasizing the wrist, (11) whipping extension of arm, and (12) drawthrough of hitting arm. A scale indicating the distances of BAC representations in movement memory is located on the left side of the figure. The lower the value of a horizontal connection between two BACs, the lower the distance between them in movement memory (Printed from: Schack, 2004a; p. 417 with permission).

To illustrate our approach, consider our recent research in professional volleyball which addressed the spike (i.e., attack hit). This movement requires at least 12 sub-steps (BACs). In preparation for a motor imagery training program, we studied this structure in the members of a Women’s Volleyball Youth National Team. Figure 3 illustrates the results for two players who are both outside hitters.

Player A (Figure 3A) was highly skilled in performing the spike movement such that she was able to optimally execute the technique. Accordingly, she held a clearly structured, almost ideal movement representation in her movement memory. BAC 1–3 in connection with 4 and 5 form the *run-up* phase. Concepts 6, 7, and 8 combine for the *hit-preparation* phase, whereas 9, 10, and 11 make up the *hit*-phase.

In contrast, player B (Figure 3B) had difficulties in optimally executing the spike for several years. The SDA-M analysis showed a problematic structure in the mental movement representation: BAC 1–3 and 4–5, which are important for the sequence of impulses during run-up and take-off, point to a less precise memory structure. For this player, run-up and take-off were broken down into two inefficient memory sections (5–2 and 4–3, encircled in Figure 3B). Subsequently, an individualized motor imagery training program for player B tackled the memory structure and developed motor imagery for an ideal take-off and a proper spike. Additionally, player B went through a series of run-up and take-off drills designed to bring out the optimal motion sequence. The focus was on making the player aware of the altered movement so that she could develop a new feeling for it. We subsequently aimed to generate this optimal *perception* of the movement also in the complementary motor imagery training. This succeeded in improving player B’s spike significantly, and she is now a member of the Women’s A-National Team. The advantage of this combination of motor imagery training and memory analysis is that athletes’ memory structures are integrated into the motor imagery training considering their individual dispositions.

Holmes and Collins (2001) made an important step towards individualized motor imagery training. These authors proposed the so-called PETTLEP approach (Physical, Environment, Task, Timing, Learning, Emotion and Perspective) to motor imagery which stresses the need for functionally equivalent and therefore behaviorally matched imagery interventions as opposed to traditional imagery interventions (e.g., Holmes and Collins, 2001; Smith et al., 2007; Wakefield et al., 2013). That is, the seven PETTLEP aspects of a given movement should be optimized in the sense that they converge between actual and imagined movement execution. This approach is partly rooted in Lang’s bio-informational theory (Lang, 1979, 1985) which states that motor imagery training affects motor performance by way of the strengthening of memory representations. Specifically, during imagery, stimulus, response, and meaning propositions^3^ are being accessed and thus strengthen the representation of the movement stored in memory, which in turn affects motor performance. Based on findings from neurophysiological research that similar processes are activated during imagined and actual motor actions, Holmes and Collins (2001) suggested that functional equivalence is a major prerequisite for the efficacy of mental practice. Therefore, behavioral matching of the imagined experience to the actual physical experience has been suggested to enhance the efficiency of motor imagery training as it is proposed to best access the underlying motor representation (Holmes and Collins, 2001; Wakefield et al., 2013).

Whereas the PETTLEP approach draws on the matching of the imagined and the actual experience in order to best access the underlying motor representation during motor imagery training, MTMR addresses the mental representation itself as the basis for motor imagery training. That is, a particular movement and its structure are emphasized in MTMR and corrected, if necessary. In that sense, the imagined movement is individually adapted, not only the embedding aspects such as the PETTLEP elements should be optimized. Lang’s (1979, 1985) bio-informational theory points to a potential mechanism of how MTMR (employing the SDA-M method) may lead to performance improvements. By emphasizing specific movement phases during motor imagery training, access or retrieval of response and meaning proposition might be facilitated and, thereby, help to improve the structure of the movement representation. It is important to note that the SDA-M method if used in motor imagery training is focused on imagining the movement in its biomechanically and functionally optimal structure.

Motor imagery training is sometimes employed using the SDA-M method by various professional and amateur sports athletes and also in rehabilitation (Braun et al., 2006, 2007; Holmes, 2007; Malouin and Richards, 2013; Malouin et al., 2013 for review) although imagery might be more efficient for stroke patients in chronic stages (Ietswaart et al., 2011). In cases of injury, motor imagery training offers a means of training even when active movement execution is severely impaired. As a result, new opportunities for motor imagery training open up in the fields of medical and orthopedic-traumatologic rehabilitation. Motor imagery training seems to be of great use for regaining lost movement patterns after joint operations (Braun et al., 2006; Holmes, 2007; Malouin et al., 2013). This provides more evidence that motor imagery training provides a general means to link imagery and movement in various areas of life.

## Empirical evidence: motor imagery training studies

Velentzas (2010) recently explored the effects of MTMR on volleyball spike performance, and on participants’ mental representations of movements. Specifically, the effects of MTMR and generic imagery scripts were investigated. Expert female volleyball players who play the outside hitter position participated. Selected movement characteristics were measured, and mental representations for these movements were evaluated using the SDA-M method. Participants’ spike accuracy was also evaluated. To control for participants’ imagery ability, the Movement Imagery Questionnaire-Revised (MIQ-R; Hall and Martin, 1997) was used. Results showed an increased performance in the post and retention test for participants in the individualized imagery script group compared to the generic script group. This result suggests that an individualized imagery script which is based on participants’ mental representations is more effective than a traditional, generic motor imagery.

Recently, we examined the influence of motor imagery training on the development of mental representation structure in early skill acquisition (Frank et al., 2014). Based on the previous finding (Frank et al., 2013) that mental representation structures functionally adapt during physical practice (i.e., during motor learning), we investigated whether mental practice adds to this adaptation process. For this purpose, novices practiced the golf putt either mentally, physically, or in a combination of both over three days, while a control group did not practice at all. Participants’ putting performance and mental representation structures (SDA-M) were tested before and after the intervention and after a retention interval of 72 h. Analyses revealed functional adaptations in mental representation structure together with improvements in putting performance for all groups. Moreover, participants who practiced mentally, either solely or in combination with physical practice, revealed representation structures that were more similar to that of experts than participants who did not practice mentally. This was the case for both, the post-test and the retention-test. These findings support the idea that mental practice in the sense of motor imagery training is beneficial to the cognitive adaptation process during motor learning.

An interesting issue to address in future studies is that of an individual’s imagery ability and its relation to the underlying mental representation of a particular motor action in memory. Imagery ability pertains to an individual’s general capability to generate and to control an image (for an overview on imagery ability, see e.g., Morris et al., 2005; Cumming and Williams, 2012) and has been found to moderate the influence of motor imagery rehearsal on performance (e.g., Goss et al., 1986; Robin et al., 2007). In this respect, a valuable objective for future research would be to explore the relationship between imagery ability, as measured by the MIQ-R (Hall and Martin, 1997), the revised version of the Vividness of Movement Imagery Questionnaire (VMIQ-2; Roberts et al., 2008), or the Sport Imagery Ability Questionnaire (SIAQ; Williams and Cumming, 2011), and mental representation, as measured by SDA-M in more detail. To explain, although holding the same level of general imagery ability, two individuals may differ on how elaborate their underlying representation of a certain motor skill is (and vice versa). Furthermore, it will be interesting to investigate whether and how MTMR affects imagery ability. Although it is well-known that motor imagery training in general can improve imagery ability (e.g., Rodgers et al., 1991), research has yet to be carried out to investigate the specific influence of MTMR on imagery ability.

## Potential relevance for technical fields

An important reason for the new interest in a cognitive-perceptual and architectural understanding of action is the impressive development of cognitive robotics. More research efforts are needed to understand how mental imagery and its mechanisms in human cognition can be applied to enhance motor control. Computational models of various kinds provide starting points to transfer the insights from the role of mental representations and motor imagery training to technical systems to enhance technical motor control in human machine interactions such as humanoid robots. Such computational models are often biologically inspired, that is, they are artificial neural nets (e.g., WALKNET, Cruse et al., 1998; Cruse and Schilling, 2013; Schilling et al., 2013 or echo state networks, Krause et al., 2010). Other cognitive-inspired computational modeling approaches of mental imagery are based on eye-movement research (Farah, 1984; Essig et al., 2012; Sima and Freksa, 2012). Such modeling approaches can reveal engineering principles for the development of autonomous systems that are capable of exploiting the characteristics of mental imagery to interact more efficiently and smoothly with the environment. Furthermore, computational models of motor control can provide novel frameworks for the question of how the central nervous system conjoins sensory signals, mental imagery and motor commands.

## Conclusions

Many theories assume that human action representations functionally integrate motor information and information on action effects. Specifically, perceptual-cognitive approaches claim that motor control comprises representations of target objects, movement characteristics, goals and anticipated disturbances. Here, we have presented a method to objectively evaluate the structure among basic action concepts, the fundamental building blocks of movement representations at the mental level. Reported evidence shows that the structure of movement representations as assessed with the SDA-M is associated with individual skill levels, biomechanical and task constraints and changes through (mental) training. Thus, it is suggested that learning progress can also be monitored by means of the SDA-M method which is an objective way to measure cognitive (movement) representations.

We have reviewed a number of studies that demonstrate the successful application of the SDA-M in professional sports such as golf, dance and volleyball and also in other settings such as rehabilitation after impairments. As the SDA-M permits a reliable, individual diagnostics of movement representations, it provides a valuable tool for individualized motor imagery training and coaching.

The methods presented here make it possible to take the essential information on the underlying cognitive-perceptual action system into account and, thereby, address the individual needs of an athlete in a better way, for example, by using the described Motor imagery Training based on Mental Representation method (MTMR). The theoretical perspective on the construction of action developed here (cf. Schack, 2004a, b), and the SDA-M method could be relevant for optimizing the daily work of the sport psychologist and also for opening up new perspectives to modify approaches to motor imagery training (Schack and Bar-Eli, 2007; Schack and Hackfort, 2007).

### Conflict of interest statement

The authors declare that the research was conducted in the absence of any commercial or financial relationships that could be construed as a potential conflict of interest.

## Footnotes

^1^According to the Schmidt’s Schema Theory, each skill action we have learned needs its own individual motor program stored in LTM. Schmidt suggested that we need 3 things to perform a skill: A generalized motor program as the basic form of our movements, like a forehand drop shot that can generate a variety of similar actions under different circumstances, e.g., shots at a variety of heights. A Recall Schema to adjust the generalized motor program for a particular action (e.g., a forehand drop at a particular height) after understanding the actual situation (initial conditions) and the intentions (response specification). When the movement is performed, the sensory consequences (e.g., feel of the movement) and response outcomes (e.g., flight path of the shuttlecock) are stored in memory. This is the so called Recognition Schema used to evaluate the outcome of the movement (response outcome) and to detect errors. If the response outcome is not perfect, the schemas are modified based on sensory feedback and knowledge of results. This leads to further adjustments of the generalized motor program when practicing a movement in order to subsequently achieve the desired outcome.

^2^Although we focus in this paper on motor imagery rehearsal, the MTMR approach is not restricted to this technique. In the MTMR approach other techniques such as relaxation, cognitive or emotional preparation can be included.

^3^According to Lang (1977, 1985), images are functionally organized sets of propositions. Stimulus propositions reflect the content of the image, response propositions the person’s responses to the image, and meaning propositions the interpretations of the image, i.e., its meaning for the person. Response proposition provide a access to the motor command system in order to generate movements. Note that these proposition are conceptualized as having an amodal format (Lang, 1977; Callow and Hardy, 2005).
